# Membrane progesterone receptors mediate progesterone-stimulated glycogenolysis in the bovine uterine epithelium

**DOI:** 10.1530/REP-24-0174

**Published:** 2024-10-18

**Authors:** Malia D Berg, Matthew Dean

**Affiliations:** 1 Department of Animal Sciences, University of Illinois at Urbana-Champaign, Urbana, Illinois, USA; 2 Division of Nutritional Sciences, University of Illinois at Urbana-Champaign, Urbana, Illinois, USA

## Abstract

In livestock, the amount of glucose needed by the endometrium and embryo increases during early pregnancy. Yet, how glucose concentrations in the endometrium are regulated remains unclear. The bovine uterine epithelium can store glucose as glycogen, and glycogen content decreases in the luteal phase. Our objective was to elucidate the role of progesterone in glycogen breakdown in immortalized bovine uterine epithelial (BUTE) cells. After 48 h of treatment, progesterone decreased glycogen abundance in BUTE cells (*P* < 0.001) but did not alter glycogen phosphorylase levels. RU486, a nuclear progesterone receptor (nPR; part of the PAQR family) antagonist, did not block progesterone’s effect, suggesting that progesterone acted through membrane progesterone receptors (mPRs). RT-PCR confirmed that BUTE cells express all five mPRs, and immunohistochemistry showed that the bovine uterine epithelium expresses mPRs *in vivo*. An mPRα agonist (Org OD 02-0) reduced glycogen abundance in BUTE cells (*P* < 0.001). Progesterone nor Org OD 02-0 affected cAMP concentrations. Progesterone increased phosphorylated AMP-activated protein kinase (pAMPK) levels (*P* < 0.001), indicating that progesterone increases intracellular AMP concentrations. However, AMPK did not mediate the effect of progesterone. AMP allosterically activates glycogen phosphorylase, and D942 (which increases intracellular AMP concentrations) decreased glycogen abundance in BUTE cells. A glycogen phosphorylase inhibitor partially blocked the effect of progesterone (*P* < 0.05). Progesterone and Org OD 02-0 had similar effects in Ishikawa cells (*P* < 0.01), a human cell line that lacks nPRs. In conclusion, progesterone stimulates glycogen breakdown in the uterine epithelium via mPR/AMP signaling. Glucose released from glycogen could support embryonic development or be metabolized by the uterine epithelium.

## Introduction

The bovine preimplantation embryo and uterine endometrium need increasing amounts of glucose during early pregnancy (Chen & Dean 2023). After fertilization, the embryo primarily uses lactate for energy, but glucose uptake and metabolism increase dramatically as the morula enters the uterine lumen (Gardner *et al.* 1993, Malkowska *et al.* 2022). Sheep blastocysts take up 50 fold more glucose than cleavage-stage embryos (Gardner *et al.* 1993). Besides glucose, the developing embryo also increases the uptake of fructose and lactate, both of which originate from maternal glucose (Gardner *et al.* 1993, Khurana & Niemann 2000, Moses *et al.* 2022, 2024*a*). However, excessive glucose negatively affects embryo development. Concentrations similar to serum concentration are too high and reduce embryo development (Kimura *et al.* 2005). Thus, glucose secretion into the uterine lumen must be tightly regulated. In support of this, glucose concentrations in uterine fluid are lower than in serum, and intravenous (IV) infusion of glucose failed to increase glucose concentrations in the uterine fluid of cows (Leane *et al.* 2018, Moraes *et al.* 2019).

The uterine epithelium also needs glucose to support pregnancy. In livestock, the epithelium can convert glucose to fructose via the polyol pathway (Steinhauser *et al.* 2016, Moses *et al.* 2024*b*). The epithelium can metabolize glucose via glycolysis, producing adenosine triphosphate (ATP) and pyruvate. Pyruvate can be further metabolized by the tricarboxylic acid (TCA) cycle and oxidative phosphorylation, yielding more ATP, or the pyruvate can be converted to lactate and released into the uterine lumen (Chase *et al.* 1992, Moses *et al.* 2024*a*). The uterine epithelium also uses glucose to glycosylate proteins. For example, the apical surface of the uterine epithelium is heavily glycosylated (Clark 2015).

The uterus does not express the enzymes necessary to make glucose *de novo* (Zimmer & Magnuson 1990, Yánez *et al.* 2003). Therefore, all glucose in the uterus must come from the maternal circulation. Glucose can enter uterine cells through facilitated diffusion (GLUTs (gene family *SLC2A)*) or be brought into cells by sodium-glucose linked transporters (SGLTs [gene family *SLC5A)*). Expression and localization of these transporters play a role in glucose uptake, metabolism, and secretion (Frolova & Moley 2011). A study found that *SLC2A1* (GLUT1) and *SLC5A1* (SGLT1) expression was lower in the endometrium of sheep treated with progesterone and a progesterone receptor antagonist than in sheep treated with only progesterone, suggesting progesterone increased expression of these transporters (Gao *et al.* 2009). However, another study in sheep found *SLC2A1* (GLUT1) and *SLC5A1* (SGLT1) expression was not affected by exogenous progesterone (Hoskins *et al.* 2021). Similarly, in cattle, expression of *SLC5A1* (SGLT1) in the endometrium increased between days 7 and 13 of the cycle and of pregnancy, and exogenous progesterone had no effect (Forde *et al.* 2010).

Progesterone plays an important role in regulating glucose uptake and metabolism in the uterus. In heifers, progesterone concentrations on day 14 of the estrous cycle and glucose concentrations in the uterine fluid were positively correlated (Simintiras *et al.* 2019*a*). Similarly, multiple studies in sheep and cattle have shown that progesterone supplementation increased the glucose concentration in uterine fluid (Hugentobler *et al.* 2010, Satterfield *et al.* 2010, Simintiras *et al.* 2019*b*). It is unclear how progesterone increases glucose concentrations in uterine fluid, given its lack of effect on glucose transporters (Forde *et al.* 2010, Hoskins *et al.* 2021).

The uterus can also regulate glucose metabolism and release by storing glucose as glycogen. Glycogen is a macromolecule composed of tens of thousands of glucose residues joined by α(1→4) and α(1→6) linkages. When glucose is in excess, it can be added to a preexisting glycogen molecule by glycogen synthase. When glucose is needed, glucose-1-phosphate is liberated from glycogen by the enzyme glycogen phosphorylase.

Glycogen is present in the endometrium of many species, including cattle, mice, rats, and humans (Dean 2019). Our lab has recently shown that in mice, the glycogen content of the uterine epithelium is high at proestrus and decreases during the preimplantation period (Chen *et al.* 2022). In cattle, the glycogen content of the glandular and luminal epithelium was lower on day 11 than on day 1 of a non-pregnant cycle (Sandoval *et al.* 2021). In cattle, progesterone concentrations are at their nadir on day 0 of the cycle and steadily increase, reaching peak concentrations on days 10–15 (Donaldson *et al.* 1970). Hence, progesterone-stimulated glycogenolysis may play a role in providing glucose to endometrial tissue or the growing embryo.

Classically, progesterone regulates uterine function by binding to one of two nuclear progesterone receptors (mPRs) (nPR-A and nPR-B). At estrus, nPRs are highly expressed in the luminal and glandular epithelium, with some expression in the stroma. As progesterone levels increase after ovulation, nPR expression in the epithelium decreases. Expression in the stroma remains the same. Confirming the decrease in nPR in the epithelium is due to the effects of progesterone, exogenous progesterone has a similar effect (Bazer *et al.* 2009, Okumu *et al.* 2010).

The uterus also expresses two classes of membrane mPRs: progesterone receptor membrane components (PGRMCs) and membrane mPRs (Moussatche & Lyons 2012). PGRMCs (genes *PGRMC1* and *PGRMC2*) are thought to act as adaptor proteins in progesterone signaling, but the precise roles of PGRMCs have not been fully elucidated (Cahill 2007, Thomas *et al.* 2007). Membrane mPRs belong to the progestin and adiponectin receptor (PAQR) family and are 7-transmembrane receptors (Tang *et al.* 2005). There are 5 mPRs, each encoded by a different gene (mPRα (*PAQR7)*, mPRβ (*PAQR8*), mPRγ (*PAQR5*), mPRδ (*PAQR6*), and mPRε (*PAQR9*)). Most research into their function has been done in oocytes and neurons; however, mPRs are known to be expressed in the uteri of humans, cattle, and dogs (Fernandes *et al.* 2005, Kowalik *et al.* 2019, Kazemian *et al.* 2023). Most frequently, they signal through cAMP, but other pathways activated by mPRs are still being uncovered (Tang *et al.* 2005, Thomas 2008).

The objectives of the current study were to (1) determine if progesterone reduces glycogen abundance in immortalized bovine uterine epithelial (BUTE) cells, (2) determine which type of progesterone receptor mediates the effects of progesterone, and 3) elucidate the intracellular pathway mediating the effects of progesterone/mPR signaling.

## Materials and methods

### Cell culture

BUTE cells were generated from the endometrium of a Holstein dairy cow at the University of Illinois Dairy Farm by our laboratory. They have been previously validated and described (Berg *et al.* 2022). Briefly, an endometrial biopsy was collected on day 1 of the cycle, digested into a single-cell suspension, plated in a 6 well plate, and transfected with a pW2 plasmid containing large T and small T antigens. Cells were passed to 10 cm plates; individual colonies with a cobblestone morphology were collected and expanded. A clone expressing keratin, lacking vimentin, with a clear cobblestone morphology, and stable growth was selected for further use. BUTE cells were confirmed to express major glycogen-metabolizing enzymes, estrogen receptor α, nPR-A, and nPR-B. They express *WNT7A* but not *FOXA2*, indicating that they represent the luminal epithelium (Gonzalez *et al.* 2022).

BUTE cells were grown in α-minimum essential media (αMEM) containing 5.55 mM glucose (complete composition in Supplementary Table 1, see section on supplementary materials given at the end of this article) supplemented with 10% fetal bovine serum (FBS; 12103C-500ML, Sigma-Aldrich), 2 mM L-glutamine (GLL01-100ML, Caisson Labs), 10 mg/mL insulin-transferrin-selenium (ITS; 25-800-CR, Corning), 1.8 ng/mL EGF (20053100UG, Shenandoah Biotechnology), and 18.2 ng/mL estradiol-17β (E2758-1G, Sigma). This glucose concentration (5.55 mM) is close to, but slightly above, physiological concentrations in cattle (Mohammed *et al.* 2021). Ishikawa cells were grown in DMEM/F12 media with 14.5 mM glucose (complete composition in Supplementary Table 2) supplemented with 10% charcoal-stripped FBS. Both cell lines were maintained at 37°C in a humidified incubator with 5% CO_2_. The use of 37°C was done to allow for the simultaneous growth of both cell lines, though 37°C is slightly below the normal body temperature of cattle (Godyń *et al.* 2019). Cell lines were passaged every 3–4 days.

### Cell culture experiments

BUTE and Ishikawa cells were plated and allowed to grow until 80% confluent. Then the media was removed, rinsed twice with phosphate-buffered saline (PBS), and the cells were serum and steroid-starved in media without phenol red and treated with insulin-ike growth factor 1 (IGF1, 50 ng/mL; 2000550UG, Shenandoah Biotechnology) for 24 h to stimulate glycogenesis (Gonzalez *et al.* 2022). The media was removed and replaced with serum and steroid-free media containing 50 ng/mL of IGF1 and appropriate treatments for indicated times. Controls were treated with a vehicle (DMSO) for all experiments. The length of treatment, number of replicates, and purpose of each experiment are outlined in Supplementary Table 3.

### Hormone, activators, and inhibitors

IGF1 (2000550UG, Shenandoah Biotechnology) was dissolved in sterile PBS at 100 μg/mL. To generate a dose response, progesterone (P8783-5G, Sigma Aldrich) and Org OD02-0 (mPRα agonist; 2085, Axon Med Chem, VA, USA) were dissolved in dimethyl sulfoxide (DMSO) at 100 mM and then diluted to 0.1, 1, and 10 mM in DMSO. RU486 (nPR antagonist; 10006317, Cayman Chemical Company, MI, USA), forskolin (adenylyl cyclase activator; 11018, Cayman Chemical Company), D942 (increases intracellular AMP; 14741, Cayman Chemical Company), A-769662 (direct activator of AMP-activated protein kinase (AMPK); 11900, Cayman Chemical Company), dorsomorphin (AMPK inhibitor; 14741, Cayman Chemical Company), and glycogen phosphorylase inhibitor (GPI; 17578, Cayman Chemical Company) were dissolved in DMSO. The final concentration of compounds in media consisted of 0.1, 1, or 10 µM for progesterone and Org OD02-0; 10 µM for RU486, forskolin, D942, A-769662, and GPI; and 5 µM for dorsomorphin. DMSO was used as the vehicle control for all experiments. At the end of each experiment, cells were confirmed to look healthy, and the monolayer was not disrupted (not shown).

### Western blot

BUTE and Ishikawa cells were plated in a 10 cm dish and treated as indicated. At the conclusion of the treatment, media was removed from each 10 cm plate, rinsed once with PBS, lysed in radioimmunoprecipitation assay (RIPA) buffer containing protease and phosphatase inhibitors (P0044-1ML, Sigma-Aldrich, and A32953, Thermo Scientific) and stored at −20°C. Protein concentration was determined with the Pierce™ BCA Protein Assay Kit (23227, Thermo Scientific). Western blots were carried out as previously described (Sandoval *et al.* 2021, Ziv-Gal *et al.* 2021, Gonzalez *et al.* 2022). Protein (25–35 µg) was loaded on 10% SDS-PAGE gels and run until adequate separation was achieved at 120 V. The gel was then transferred onto a Polyvinylidene fluoride (PVDF) membrane. Membranes were blocked with 5% BSA or 5% powdered milk in tri-buffers saline (TBS) with 0.1% tween (TBS-T). The primary antibodies (Supplementary Table 3) were diluted in block (Supplementary Table 4), added to the membranes, and incubated overnight at 4°C. After three washes with TBS-T, the appropriate secondary antibody (anti-rabbit; 7074S, Cell Signaling) in the sample block was added to the membrane and incubated for 30 min. All blots were developed using SuperSignal West Pico PLUS chemiluminescent substrate (34577, Thermo Scientific) and imaged using the FluorChem M (Protein Simple, CA, USA).

### Immunohistochemistry

Uteri from cows (*n* = 4) were collected on days 1 and 11 of the estrous cycle, fixed, and placed into paraffin blocks as part of a previous project (Sandoval *et al.* 2021). Paraffin blocks were sectioned at 5 µm, and two to three sections were placed on a slide and allowed to dry overnight. Slides were deparaffinized and rehydrated. Slides were placed in 0.01 M sodium citrate (pH 6.0) and microwaved twice for 5 min to retrieve the antigen. The slides were briefly placed in TBS to ensure they were at room temperature, followed by 3% hydrogen peroxide (H325-500, Fisher Scientific) in deionized water for 15 min. The slides were placed into a hydrated chamber, and a block (3% BSA, 10% goat serum, in TBS) was added to the slides for 1 h at room temperature.

The primary antibodies are listed in Supplementary Table 4. The antibody against mPRα was previously validated (Kowalik *et al.* 2019). Here, the antibodies for mPRδ and mPRε were validated using a blocking peptide (Supplementary Figure 1A). Antibodies were incubated overnight at 4°C with their protein target. The next day, the antibodies were used for immunohistochemistry as described. A lack of signal in the tissue indicated that the antibody had bound to the recombinant protein.

Antibodies were added to the slides and incubated at 4°C overnight. The slides were washed in TBS-T, then an anti-goat secondary antibody (BA-5000, Vector Laboratories) was added for 30 min at room temperature, and the ABC complex (PK-4000, Vector Laboratories) was prepared. The slides were washed, and the ABC complex was added to the slides for 30 min. After the final wash, the slides were placed on white bench paper to witness the DAB reaction (SK-4100, Vector Laboratories). The slides were counterstained with hematoxylin, dehydrated, and placed in xylene overnight prior to being mounted with Permount (SP15100, Fisher Scientific) the next day. Images were captured with a Zeiss Axioskop upright microscope with an Axiocam 305 color camera. Negative control slides were processed the same, except the primary antibody was replaced with an isotype-specific negative control (anti-GFP antibody; 2956 Cell Signaling).

### Glycogen assay

Intracellular glycogen was measured as previously validated by our laboratory (Berg *et al.* 2022, Gonzalez *et al.* 2022). At the end of treatment, BUTE and Ishikawa cells were collected with trypsin, counted, centrifuged at 4°C, and resuspended in 30% potassium hydroxide (KOH; 221473-500G, Sigma-Aldrich) to inactivate glycogen-metabolizing enzymes. Samples were stored at −20°C until analysis. Samples were thawed to room temperature, and a standard curve (0–200 µg/mL glycogen) was prepared. All samples, including the standard curve and blank, were incubated for 30 min at 95°C and then centrifuged for 1 min at 18,213 × ***g*** at room temperature. After, 480 µL of 100% ethanol was added to all samples and centrifuged at room temperature for 20 min at 18,213 × ***g*** to precipitate glycogen. The supernatant was aspirated, and 250 µL of 70% ethanol was added. The samples were centrifuged for 20 min at 18,213 × ***g*** (room temperature) to remove any remaining glucose. The supernatant was decanted, and the samples were dried overnight. The next day, 50 µL of hydrochloric acid (HCl; A144-500, Fisher Chemical) was added to each sample and incubated at 95°C for 3 h to hydrolyze glycogen. After 3 h, 50 µL of sodium hydroxide (NaOH; S318-500, Fisher Chemical) was mixed with the samples to neutralize acidity. Next, 40 µL of the sample was placed into a 96 well plate in duplicate. Wako Reagent (250 µL; 99703001, FUJIFILM Medical Systems USA) was added to each well and incubated for 30 min at 37°C. The absorbance was read at 505 nm using the µQuant plate reader (Biotek Instruments). The standard curve was used to determine the amount of glycogen in each sample and normalized to the number of cells.

### RNA isolation and reverse-transcriptase polymerase chain reaction

Polymerase chain reaction (PCR) was used to determine if BUTE and Ishikawa cells expressed the mPRs. Total RNA was extracted from BUTE and Ishikawa cells using TRIzol (15-596-018, Thermo Fisher Scientific). iScript™ Reverse Transcription Supermix (1708841, Bio-Rad) was used to transcribe 1 μg of RNA into cDNA for PCR. Promega GoTaq™ DNA Polymerase (PRM3005, Thermo Fisher Scientific) kit was used for each mPR PCR reaction and no-template control. Primers were designed specifically for each mPR from the *Bos taurus* and *Homo sapiens* genome (Supplementary Table 5). The thermo-cycling conditions were 95°C for 2 min followed by 95°C for 1 min, 55°C for 1 min, and 72°C for 30 s for a total of 40 cycles, then 72°C for 5 min in the T100 Thermal Cycler (Bio-Rad). The PCR products (5 µL) were loaded onto a 4% agarose gel and run for 90 min at 100 V. The gel was imaged using FluorChem M (Protein Simple, CA, USA).

### cAMP ELISA

A cyclic AMP (cAMP) ELISA kit (581001, Cayman Chemical Company) was used to determine the level of cAMP in BUTE cells. The manufacturer’s instructions were followed. Briefly, the media was aspirated, and 1.6 mL of 0.1 M HCl was added to the plate and incubated at room temperature for 20 min. The cells were scraped off the plate with a cell scraper and placed into a 2 mL microcentrifuge tube. Next, the tubes were centrifuged at 1,000 x ***g*** for 10 mi at room temperature, and the supernatant was transferred into a new 2 mL tube and stored at −80°C until all replicates were collected. The samples were thawed and 50 µL of the sample was transferred to a new tube and diluted with 100 µL of ELISA buffer. A standard curve (0.3–750 pmol/mL) was prepared. Standard, sample, and blank were added to wells in duplicate. Next, 50 µL of cAMP AChE tracer was added to each well except for the total activity (TA) and blank wells, and 50 µL of cAMP ELISA antiserum was added to each well except for the TA, non-specific binding (NSB), and blank wells. The plate was covered and incubated for 18 h at 4°C. The next day, the plate was washed five times with wash buffer, then 200 µL of Ellman’s Reagent was added to each well, and 5 µL of tracer was added to the TA wells. The plate was covered with a plastic film and then placed on a shaker with a box covering the plate to allow it to develop in the dark for 120 min at room temperature. The plate was read at 415 nm using the µQuant plate reader (Biotek Instruments). The cAMP concentration was calculated using the spreadsheet provided by Cayman Chemical Company.

### Statistical analysis

One-way ANOVA followed by Dunnett’s multiple comparisons test or Tukey’s multiple comparison test was used for all experiments with >2 treatments. Experiments with 2 treatments were analyzed using a *t*-test. Statistical analyses were conducted in GraphPad PRISM (Version 9.0.0). Statistical significance was defined as *P* < 0.05.

## Results

### Progesterone stimulates glycogen breakdown via membrane mPRs

**Figure 1:**
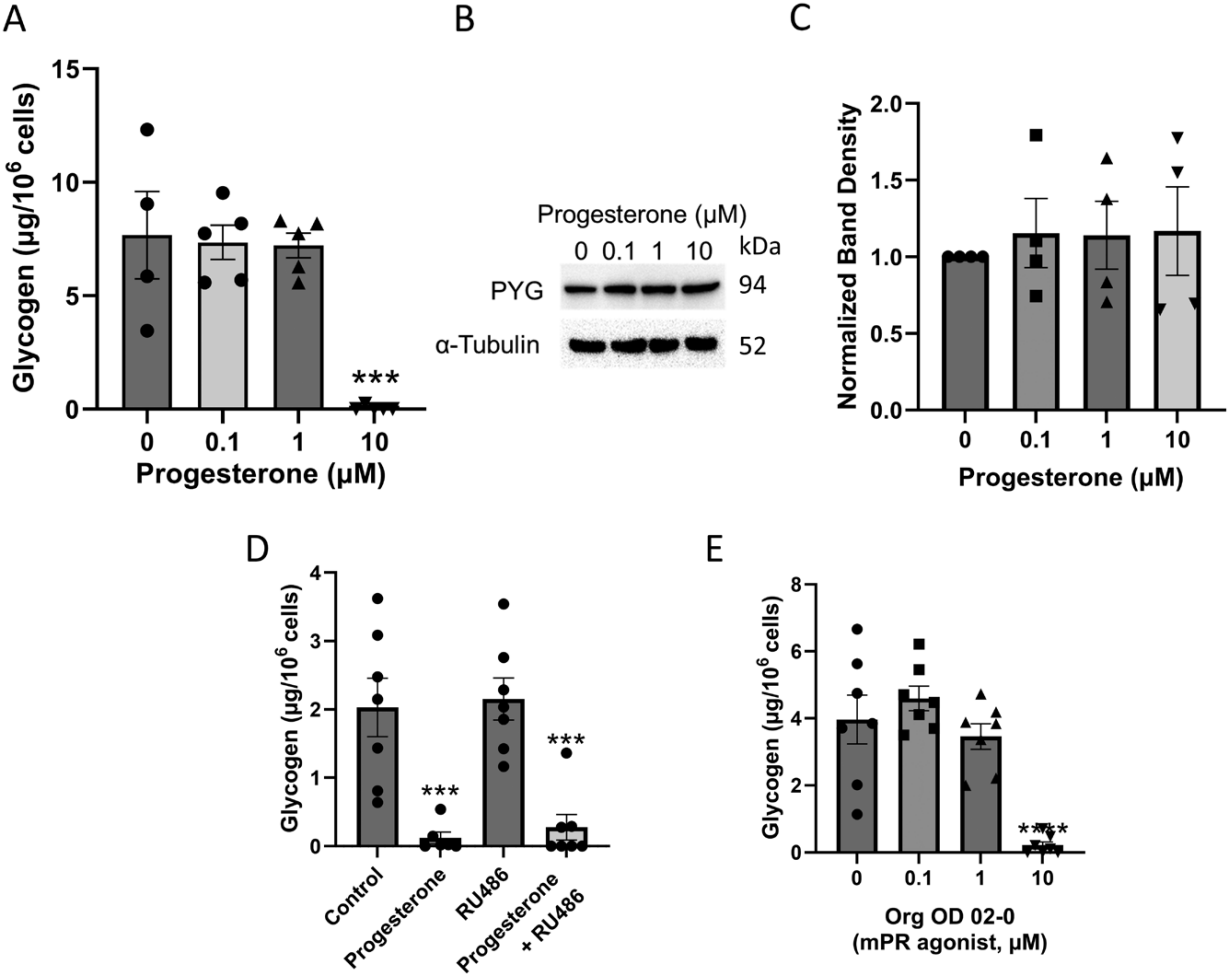
Progesterone stimulates glycogen breakdown in BUTE cells via membrane progesterone receptors (mPRs). (A) Glycogen abundance in BUTE cells treated with progesterone for 48 h (0, 0.1, 1, and 10 µM). (B) Representative western blots for glycogen phosphorylase levels in BUTE cells treated with progesterone (0, 0.1, 1, and 10 µM). (C) Quantification of glycogen phosphorylase levels normalized to α-tubulin. (D) Glycogen abundance in BUTE cells treated with 10 µM progesterone and 10 µM RU486 as indicated for 48 h. (E) Glycogen abundance in BUTE cells treated with the specific mPR agonist Org OD 02-0 (0, 0.1, 1, and 10 µM). All treatments for 48 hours. *n* = 4–6. ***P < 0.001 relative to 0 µM or control. ****P < 0.0001 relative to 0 µM. PYG, glycogen phosphorylase.

BUTE cells were treated with a range of progesterone concentrations (0–10 µM) for 48 hours to determine if there was a dose-dependent effect. Surprisingly, 0.1 and 1 µM had no effect, but 10 μM dramatically decreased the abundance of glycogen in BUTE cells by 99% compared to the 0 µM (Fig. 1A; *n* = 4–5; *P* < 0.001). To determine if progesterone was stimulating glycogenolysis by increasing glycogen phosphorylase levels, we measured glycogen phosphorylase by western blot. However, progesterone had no effect on protein levels (Fig. 1B and C; *n* = 4).

We have previously shown that BUTE cells express nPRs (PR-A and PR-B) (Berg *et al.* 2022). Here, PCR showed that BUTE cells expressed both *PGRMC* genes and all five mPR genes (Supplementary Figure 1B). To determine which type of progesterone receptor was mediating the effect of progesterone, BUTE cells were treated with progesterone and RU486, which antagonizes nPRs and PGRMCs (Baird 1993, Zheng *et al.* 2017). As expected, progesterone dramatically decreased the amount of glycogen in BUTE cells relative to the vehicle control (*P* < 0.001; *n* = 6–7), and RU486 had no effect. In cells treated with progesterone + RU486, glycogen decreased 86% compared to the vehicle, similar to the effect of progesterone alone (Fig. 1D; *P* < 0.001 compared to control). Thus, we hypothesized that mPRs were mediating the effect of progesterone. To test this hypothesis, we used Org OD 02-0, an mPRα agonist (Kelder *et al.* 2010). BUTE cells were treated with 0, 0.1, 1, and 10 µM of the mPRα agonist Org OD 02-0 for 48 h. BUTE cells treated with 10 µM Org OD 02-0 had a 94% decrease in glycogen abundance compared to the vehicle control (Fig. 1E; *n* = 7; *P* < 0.0001). These results indicate that progesterone stimulates the breakdown of glycogen in BUTE cells by activating the mPRs.

**Figure 2:**
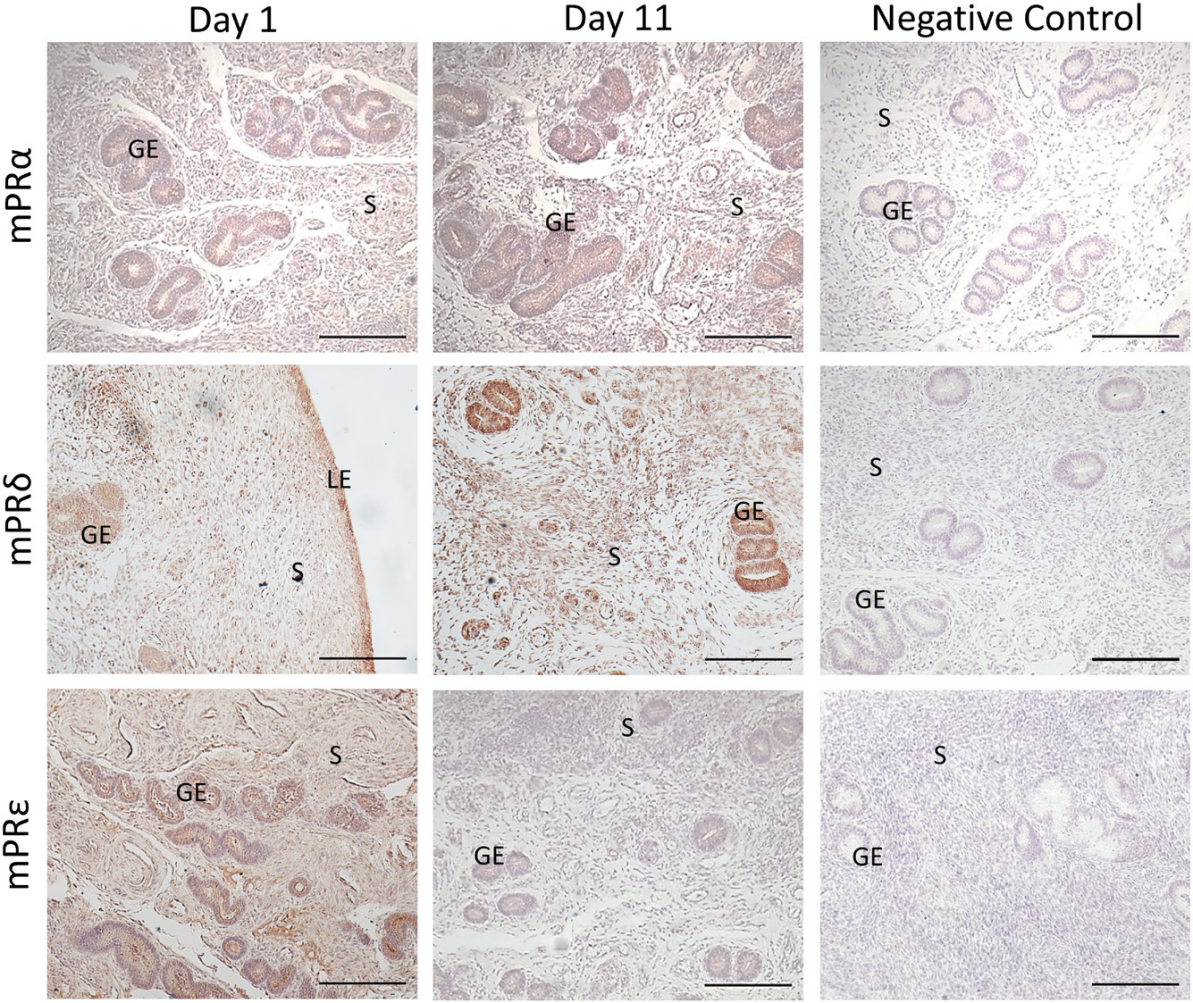
*In vivo*, the bovine endometrium expresses mPRs. Immunohistochemistry for mPRα, mPRδ, and mPRε in the cow endometrium on Days 1 and 11 of the cycle. GE, glandular epithelium; LE, luminal epithelium; S, stroma; *n* = 4. Scale bar = 200 µm. Negative control was an iso-type matched anti-GFP antibody.

For mPRs to mediate the effect of progesterone, mPRs would have to be expressed *in vivo*. Immunohistochemistry confirmed that mPRα, mPRδ, and mPRε are highly expressed in the glandular epithelium (*n* = 4; Fig. 2). mPRα and mPRδ were expressed in the epithelium and stroma. Immunostaining was moderately more intense in the glandular epithelium than in the stroma. Immunostaining did not show any clear difference between day 1 and day 11. mPRε immunostaining in both the glandular epithelium and stroma was notably more intense on Day 1 than on Day 11 (Fig. 2). Previous research has already shown that the bovine uterine epithelium expresses mPRα, mPRβ, and mPRγ (Kowalik *et al.* 2019), indicating that the bovine endometrium expresses all 5 mPR isoforms.

### mPRs do not increase cAMP levels

**Figure 3:**
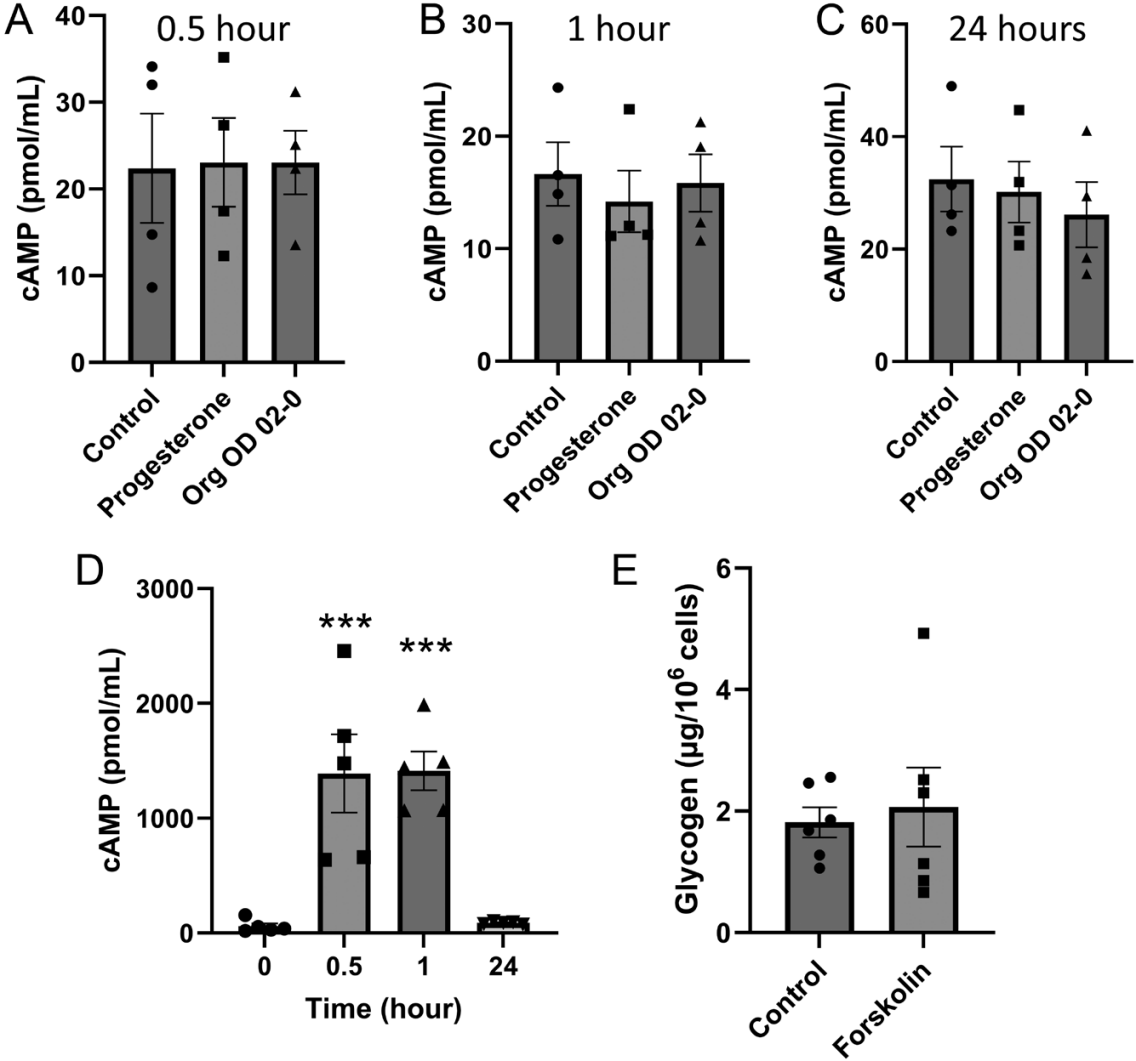
Progesterone does not regulate glycogen via cAMP. A-C) cAMP concentrations in BUTE cells treated with 10 µM of progesterone or 10 µM of Org OD 02-0 at 0.5 h (A), 1 h (B), and 24 h (C). (D) cAMP levels in BUTE cells treated with 10 µM of forskolin (adenylyl cyclase activator) at 0, 0.5, 1, and 24 h. (E) Glycogen abundance in BUTE cells treated with forskolin (10 µM) for 48 h. *n* = 5–6. ***P < 0.001 relative to 0 h.

Research in other cell types has shown that mPRs can increase cAMP concentrations (Valadez-Cosmes *et al.* 2016). However, neither progesterone nor Org OD 02-2 (both at 10 μM) changed cAMP concentrations in BUTE cells after 0.5, 1, or 24 h of treatment compared to hour 0 (Fig. 3A, B, and C; *n* = 4). To determine if cAMP could regulate glycogen, BUTE cells were treated with forskolin, an adenylyl cyclase activator. BUTE cells treated with 10 μM forskolin showed a 25 fold increase in cAMP concentrations at 0.5 and 1 h relative to hour 0 h (*P* < 0.0001; *n* = 5; Fig. 3D). However, treatment with forskolin for 48 h did not change the abundance of glycogen in BUTE cells (*n* = 6; Fig. 3E).

### AMPK is activated downstream of the mPR but does not decrease glycogen in BUTE cells

**Figure 4:**
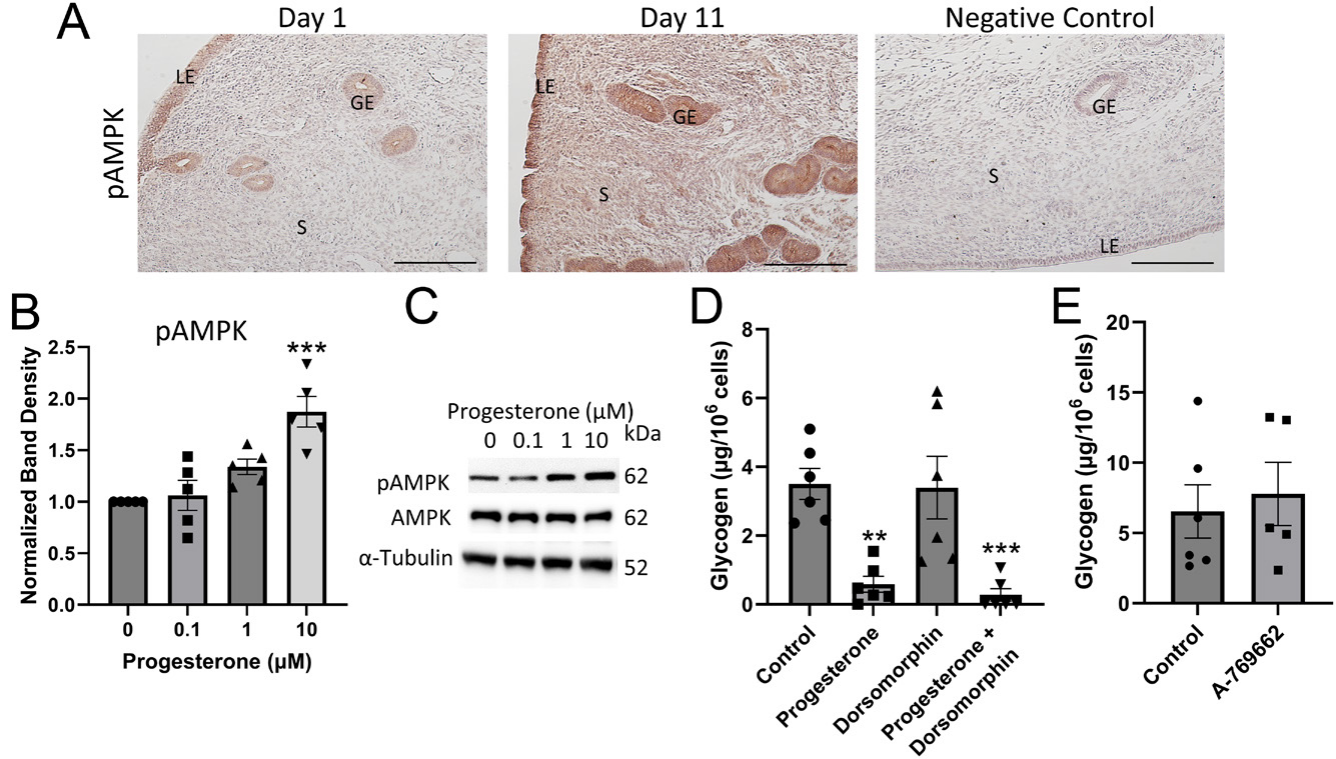
Activation of AMPK does not stimulate glycogen breakdown in BUTE cells. (A) Immunohistochemistry for pAMPK in the cow endometrium on Days 1 and 11 of the cycle. GE, glandular epithelium; LE, luminal epithelium; S: stroma. Scale bar = 200 µm. (B-C) Western blot showing the amount of AMPK and pAMPK protein in BUTE cells treated with various concentrations of progesterone as indicated for 24 h. Band density for pAMPK is normalized to α-tubulin. (D-E) Glycogen abundance in BUTE cells treated with progesterone (10 μM), an AMPK inhibitor dorsomorphin (5 µM), or AMPK activator A-769662 (10 µM) for 48 h. *n* = 4–6. **P < 0.01, ***P < 0.001 relative to control or 0 µM.

Progesterone/mPR signaling can also activate AMPK, a regulator of glycogenolysis (Nie *et al.* 2022). When intracellular AMP concentrations increase, AMP binds to AMPK, changing its conformation. This increases AMPK phosphorylation (pAMPK), and hence it is activated (Steinberg & Hardie 2022). Immunohistochemistry confirmed that pAMPK is expressed in the cow endometrium (*n* = 4; Fig. 4A). Specifically, immunostaining was darker in the glandular and luminal epithelium than in the stromal (Fig. 4A). The immunostaining was clearly more intense in the epithelium on day 11 than on day 1 of the cycle (Fig. 4A), suggesting that phosphorylation of AMPK could be regulated by progesterone. Confirming this, BUTE cells treated with 10 µM of progesterone for 24 h had an 18% increase in pAMPK levels determined by western blot (Fig. 4B and C; *n* = 5; *P* < 0.001).

To determine if the increase of pAMPK led to decreased glycogen abundance, BUTE cells were treated with progesterone and dorsomorphin, an AMPK inhibitor. BUTE cells were treated with a vehicle, progesterone (10 μM), dorsomorphin (5 μM, an AMPK inhibitor), or progesterone + dorsomorphin. However, dorsomorphin was not able to block the effect of progesterone, as progesterone decreased glycogen abundance by 92% in the presence of dorsomorphin (*n* = 6; Fig. 4D). To confirm that AMPK does not regulate glycogen abundance in BUTE cells, they were treated with a direct AMPK activator (A-769662). Treatment with A-769662 had no effect on glycogen abundance relative to control (*n* = 5–6; Fig. 4E). These results show that progesterone increases pAMPK levels, but this increase in pAMPK is not responsible for the decrease in glycogen.

### Increased AMP decreases glycogen in BUTE cells

**Figure 5:**
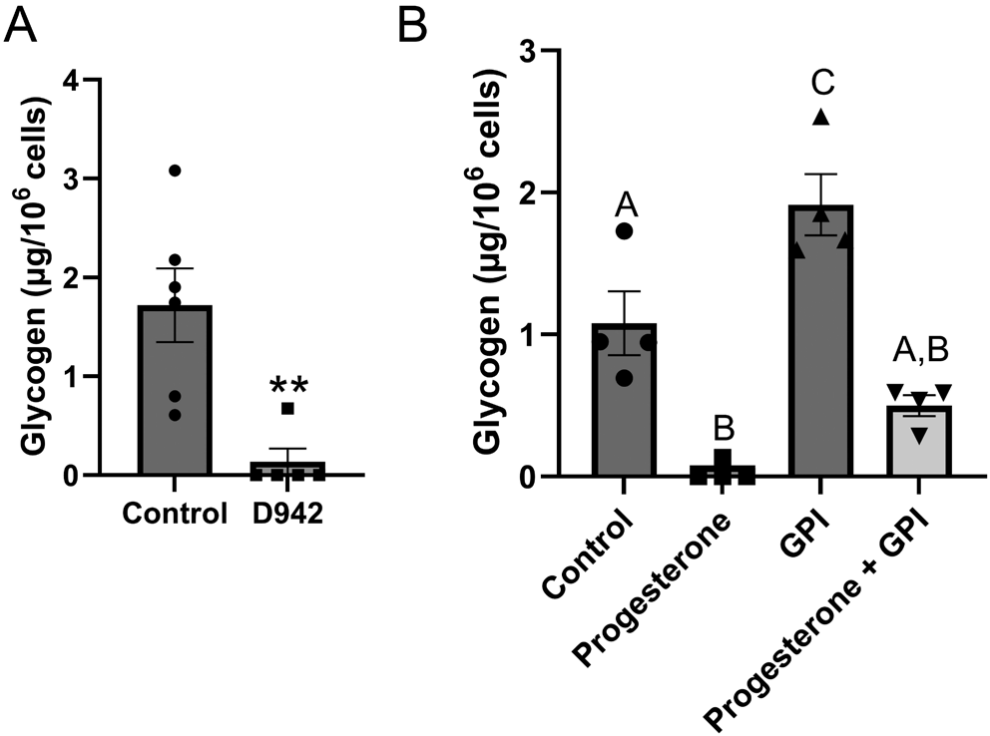
An increase in AMP stimulates glycogenolysis in BUTE cells. (A-B) Glycogen abundance in BUTE cells treated with D942 (increases intracellular AMP, 10 µM; A) or progesterone (10 µM) and glycogen phosphorylase inhibitor (GPI, 10 µM; B) for 48 hours. *n* = 6. **P < 0.01 relative to control.

AMP directly activates glycogen phosphorylase by changing the conformation of the enzyme (Agius 2015). To determine if AMP stimulated glycogen breakdown in BUTE cells, they were treated with D942 (Fig. 5A). D942 increases intracellular AMP concentrations (Kosaka *et al.* 2005). Treatment with 10 μM of D942 for 48 h reduced glycogen abundance by 92% relative to control (Fig. 5A; *n* = 5–6; *P* < 0.01).

To confirm that increased glycogen phosphorylase activity mediates the reduction in glycogen abundance induced by progesterone, we used a glycogen phosphorylase inhibitor (GPI). BUTE cells were treated with vehicle, progesterone (10 μM), GPI (10 μM), or progesterone + GPI. BUTE cells treated with GPI alone had a 77% increase in glycogen abundance compared to the control, indicating that cells have a basal level of glycogen catabolism. BUTE cells treated with progesterone had a decrease in glycogen abundance by 97% compared to control (Fig. 5B; *n* = 4; *P* < 0.01). The glycogen abundance in BUTE cells treated with progesterone + GPI was decreased by 53% compared to control cells (Fig. 5B; *P* < 0.01). This suggests that glycogen phosphorylase mediates part, but not all, of the effect of progesterone.

### Progesterone acting through the mPR stimulates glycogen breakdown in Ishikawa cells lacking nPRs

**Figure 6:**
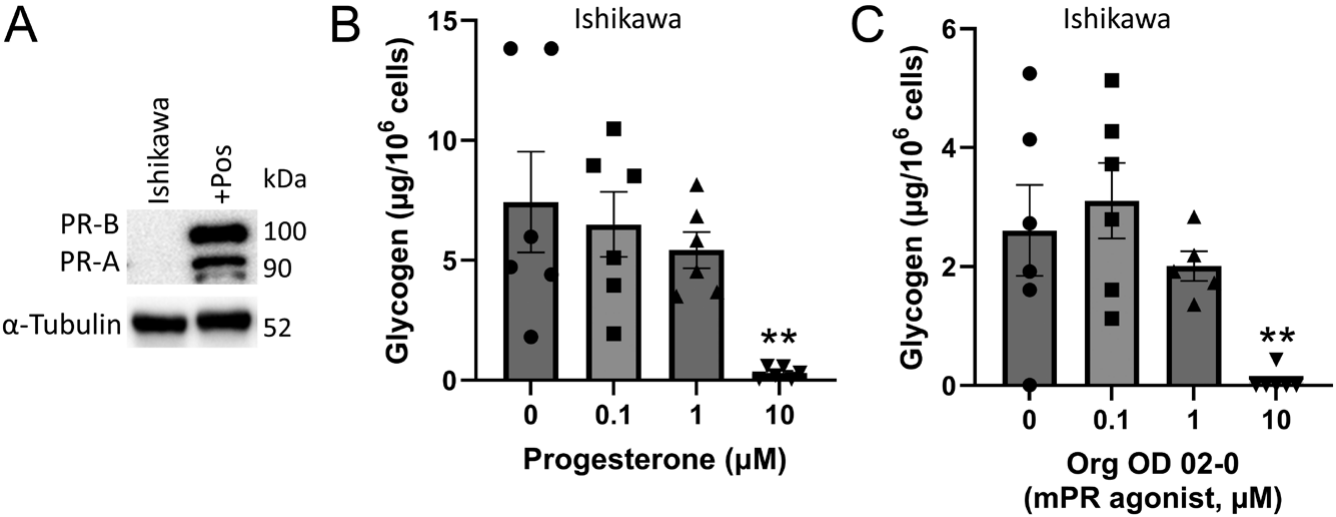
mPR stimulates glycogenolysis in human cells. (A) Western blot to show Ishikawa cells lack the nuclear progesterone receptors. (B-C) Glycogen abundance in Ishikawa cells that lack the nuclear progesterone receptor treated with progesterone (0, 0.1, 1, and 10 µM; B) or the mPRα agonist Org OD 02-0 (0, 0.1, 1, and 10 µM; (C) for 48 h. +Pos: positive control for PR-A and PR-B. *n* = 6. **P < 0.01 relative to 0 µM.

**Figure 7:**
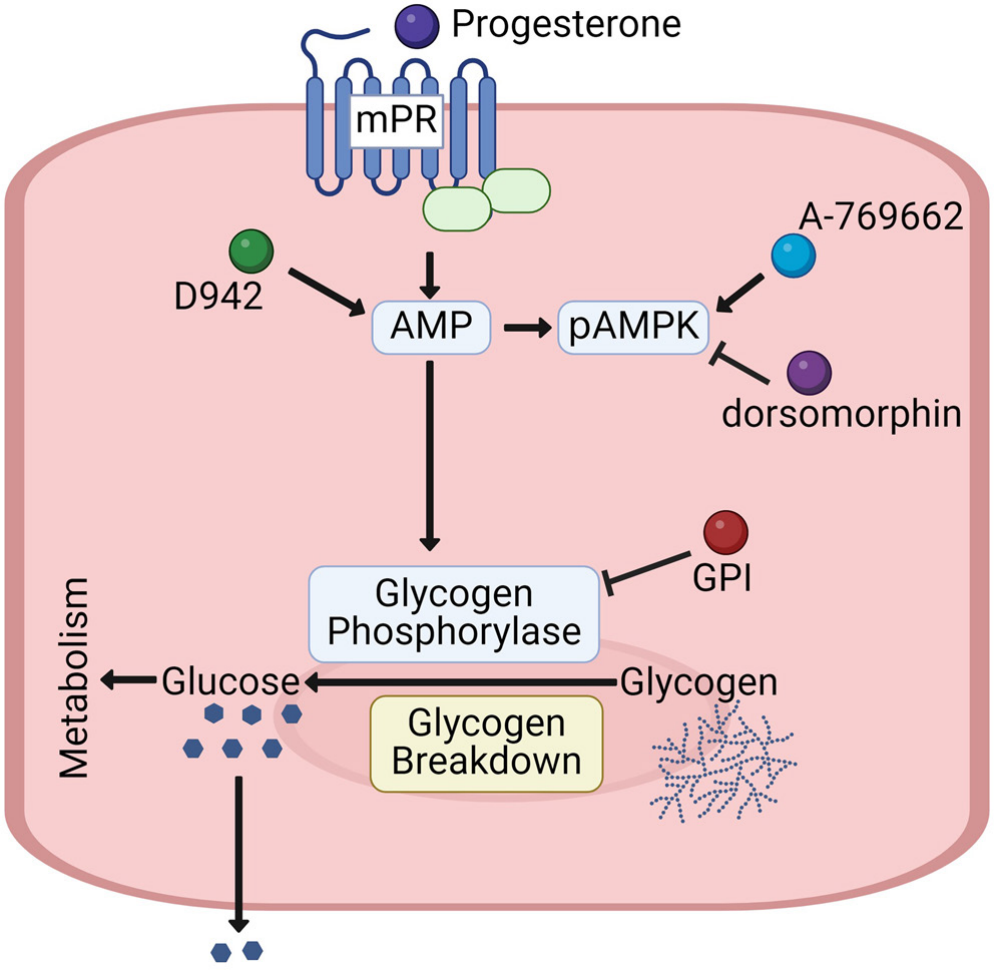
Summary of the proposed mechanism. Progesterone binds to the membrane progesterone receptors (mPR) stimulating AMP production. AMP results in phosphorylation of AMPK (pAMPK). AMP also directly activates glycogen phosphorylase, leading to glycogen catabolism. The resulting glucose can be metabolized by the epithelial cell or secreted into the uterine lumen. D942 increases AMP concentrations by inhibiting NAD(P)H dehydrogenase [quinone] 1 (complex I); A-769662, AMPK activator; dorsomorphin, AMP inhibitor; GPI, glycogen phosphorylase inhibitor.

To confirm that progesterone stimulates glycogen breakdown in other species, glycogen was measured in Ishikawa cells (human uterine epithelial cancer cell line). Ishikawa cells lack PR-A and PR-B (Dean *et al.* 2018), allowing us to also confirm that the effects of progesterone are independent of nPRs. Here, we confirmed that Ishikawa cells lack PR-A and PR-B by western blot (Fig. 6A). However, Ishikawa cells expressed mPRα (*PAQR7*), mPRβ (*PAQR8*), mPRγ (*PARR5*), *PGRMC1*, and *PGRMC2* at the mRNA level (Supplementary Figure 1C). Like BUTE cells, treating Ishikawa cells with 0.1 or 1.0 μM progesterone or Org OD 02-0 had no effect (*n* = 6; Fig. 6B and C). But in Ishikawa cells treated with 10 μM progesterone or 10 μM Org OD 02-0, glycogen abundance decreased by 96% and 97%, respectively (Fig. 6B and C; *n* = 6; *P* < 0.01). These results indicate that progesterone acts through the mPRs in uterine epithelial cells to stimulate glycogenolysis in both human and bovine models (Fig. 7).

## Discussion

We have shown that the glycogen content of the bovine uterine epithelium is high near estrus and low in the luteal phase (Sandoval *et al.* 2021). Additionally, we found that estradiol indirectly stimulates glycogenesis in uterine epithelium via IGF1 (Gonzalez *et al.* 2022). Here, we show that a high concentration of progesterone decreased glycogen abundance by acting through the mPRs and AMP in the BUTE and Ishikawa cells. Collectively, this suggests that cyclic changes in glycogen content of the uterine epithelium are regulated by ovarian steroids. In the follicular phase, glycogen is stored in response to the actions of estradiol/IGF1. This glycogen is mobilized after ovulation due to the direct effects of progesterone on the uterine epithelium.

In our *in vitro* models, it took a high progesterone concentration (10 μM) to reduce glycogen abundance and increase pAMPK levels. This is much higher than serum concentrations. Supporting the use of these concentrations, these changes mirror the decrease in glycogen content and increase in pAMPK levels we observed *in vivo*. The necessity of these high concentrations could be due to several reasons. First, the concentration of progesterone in uterine tissue is much higher than in serum, ranging from 169–297 ng/g (around 1 µM) (Weems *et al.* 1988, 1989). Secondly, BUTE and Ishikawa cells were treated with high IGF1 concentrations (50 ng/mL) while simultaneously being treated with progesterone. *In vivo*, IGF1 production would decrease while progesterone concentrations increase (McCarthy *et al.* 2012, Gonzalez *et al.* 2022). Thus, it may take higher concentrations of progesterone to overcome the effects of IGF1 used in our study. Supporting our use of 10 μM *in vitro*, many published articles use the same concentration to study the endometrium (Harduf *et al.* 2009, Jiang *et al.* 2017, Kasubuchi *et al.* 2017, Park *et al.* 2020, Long *et al.* 2021).

Previous research suggested that progesterone reduces glycogen in the uterine epithelium of other species (Nie *et al.* 2022). Yet the mechanism of how progesterone decreases glycogen had not been explored. There are several ways to decrease intracellular glycogen abundance. The first is to break down glycogen into glucose-1-phosphate by glycogen phosphorylase, and then glucose-1-phosphate is isomerized to glucose-6-phosphate. Glucose-6-phosphate can be used by the cells or dephosphorylated by glucose-6-phosphatase. We have previously shown that both of these enzymes are present in the uterine epithelium (Sandoval *et al.* 2021). Here, we found that progesterone decreased glycogen abundance without increasing protein levels of glycogen phosphorylase, suggesting that progesterone may increase glycogen phosphorylase activity. In support of this, glycogen phosphorylase is directly activated by AMP, and the phosphorylation of AMPK indicates that progesterone increased intracellular AMP concentrations. Also supporting this, GPI partially blocked the effect of progesterone.

Another way to catabolize glycogen is inside lysosomes via α-acid glucosidase. Glycogen is taken into the autophagosome, which then fuses with a lysosome to degrade the glycogen (Koutsifeli *et al.* 2022). α-acid glucosidase breaks the α-1,4 links between two glucose molecules to release the glucose from the glycogen molecule (Adeva-Andany *et al.* 2016). Once glycogen is broken down into glucose, it diffuses out of the lysosome through glucose transporters and into the cytosol. α-acid glucosidase activity has been detected in the ovine endometrium, but activity did not change throughout the estrous cycle (O’Shea & Murdoch 1978). In agreement, we recently showed that acid alpha-glucosidase (GAA) is expressed in the bovine uterine epithelium, but progesterone did not change GAA activity in BUTE cells (Berg & Dean 2024).

Finally, glycogen could be released from the cells by extracellular vesicles (EVs). Several studies in the uterus have shown that EVs are produced by the endometrial epithelium and secreted into the uterine lumen to aid in the embryo-endometrium crosstalk (O’Neil *et al.* 2020, Sui *et al.* 2023). Enzymes often physically associated with glycogen molecules have been found in EVs isolated from the uterus during pregnancy in cattle (Kusama *et al.* 2018). Two TEM studies of the human uterus appear to show glycogen being encapsulated into vesicles and secreted into the glandular lumen (Cornillie *et al.* 1985, Demir *et al.* 2002). However, more research is needed to determine if EVs do contain glycogen.

One limitation of the study was that it relied primarily on an *in vitro* model. Cell lines cannot fully recapitulate *in vivo* conditions. However, this model allowed us to examine the direct effects of progesterone on the uterine epithelium and work out intracellular signaling pathways that could not be done *in vivo*. Our major results, such as progesterone decreasing glycogen in the uterine epithelium, progesterone increasing pAMPK levels, and the uterine epithelium expressing mPRs, all agree with our *in vivo* observations (Sandoval *et al.* 2021).

No activator or inhibitor is perfect, and we used several in this work. Whenever possible, we tried to confirm our results in multiple ways. For example, we used progesterone, RU486, and Org OD 02-0 to differentiate between nPR and mPR. We also confirmed that the effect of progesterone is via mPRs in Ishikawa cells that lack nPRs. Org OD 02-0 is an mPRα agonist; however, how it interacts with the other mPRs is unknown. Given their high similarity, Org OD 02-0 likely activates the other mPRs as well. Currently, no tools are available that would allow us to efficiently identify which mPR mediates the effect of progesterone.

We used forskolin to test the effect of cAMP on glycogen. We were able to directly confirm that forskolin increased cAMP, though it did not have any effect on glycogen. To test the role of AMPK, we used both an inhibitor and an activator, which indicated that AMPK does not stimulate glycogenolysis in BUTE cells. We used D942 to increase intracellular AMP concentrations. D942 does this by partially inhibiting NAD(P)H dehydrogenase [quinone] 1 (complex I) (Kosaka *et al.* 2005). D942 is sometimes marketed as an indirect AMPK activator because increasing intracellular AMPK concentrations leads to AMPK phosphorylation. In the current study, we rule out the possibility that D942 is activating AMPK to reduce glycogen abundance by using a direct AMPK activator and an AMPK inhibitor. Finally, GPI partially blocked the effect of progesterone, suggesting other pathways may mediate the effect of progesterone. The increase in glycogen abundance due to GPI treatment alone suggests that GPI can inhibit basal glycogen phosphorylase activity.

In conclusion, we show that progesterone stimulates glycogenolysis in uterine epithelial cells, explaining the decrease in epithelial glycogen that has been observed in the luteal phase or during pregnancy of cows and other species (Bowman & Rose 2017, Dean 2019, Sandoval *et al.* 2021). Importantly, this effect of progesterone was mediated by the mPRs, the first functional effect of mPRs found in the uterus. Glucose liberated from glycogen could be diffused into the uterine lumen to support embryo development. It could also be converted to other nutrients, such as fructose or lactate, used by the embryo prior to diffusion (Moses *et al.* 2022, Chen & Dean 2023). Finally, the glucose could be used by the epithelial cells to meet their energy requirements or to produce glycans that mediate embryo-epithelium attachment at the beginning of implantation (Gonzalez *et al.* 2022, Sun *et al.* 2023). Further research needs to determine the importance of glycogen in the uterus and the fate of the liberated glucose during early embryo development.

## Supplementary Materials

Supplementary Material

## Declaration of interest

The authors declare that there is no conflict of interest that could be perceived as prejudicing the impartiality of the research reported.

## Funding

The study was funded by startup funds provided by the University of Illinois, USDA National Institute of Food and Agriculturehttp://dx.doi.org/10.13039/100005825, Hatch project ILLU-538-949, NIH grant R21HD112772, and NIH grant R01HD111706 to MD.

## Data availability

All data is available upon request.

## Author contribution statement

MD and MDB designed the experiments, and MDB carried out the experiments. MD and MDB drafted the manuscript. All authors approved the final manuscript.

## Acknowledgments

The authors would like to thank the past and current members of the Dean Lab.
