# Supplementary material for: Membrane progesterone receptors mediate progesterone-stimulated glycogenolysis in the bovine uterine epithelium
Source: Reproduction. 2024 Oct 18;168(6):e240174. doi: 10.1530/REP-24-0174 (PMC11558801; doi:10.1530/REP-24-0174)
Supplement: Supplementary Material [file supplementary_material.pdf]

Supplementary Tables

Supplemental Table 1. Composition of αMEM media.

Component	Concentration (mg/L)
2'-Deoxyadenosine	10
2'-Deoxycytidine	11
2'-Deoxyguanosine	10
Adenosine	10
Biotin	0.1
Calcium Chloride	200
Choline Chloride	1
Cytidine	10
D-Calcium Pantothenate	1
D-Glucose	1,000
Folic Acid	1
Glycine	50
Guanosine	10
L-Alanine	25
L-Arginine	127
L-Ascorbic Acid	50
L-Asparagine	50
L-Aspartic Acid	30
L-Cysteine	100
L-Cystine	31
L-Glutamine	292
L-Histidine	42
L-Isoleucine	52
L-Leucine	52
L-Lysine	73
L-Methionine	15
L-Phenylalanine	32
L-Proline	40
L-Serine	25
L-Threonine	48
L-Tryptophan	10
L-Tyrosine	52
L-Valine	46
Lipoic Acid	0.2
Magnesium Sulfate	98
Myo-Inositol	2
Niacinamide	1
Phenol Red	10
Potassium Chloride	400
Pyridoxal	1
Pyruvic Acid	110
Riboflavin	0.1
Sodium Chloride	6,800
Sodium Phosphate	140
Thiamine	1
Thymidine	10
Uridine	10
Vitamin B12	1.4
Sodium Bicarbonate	2,200

Supplemental Table 2. Composition of DMEM/F12 media.

Component	Concentration (mg/L)
Biotin	0.00365
Calcium Chloride	116.65
Choline Chloride	8.98
Cupric Sulfate, Pentahydrate	0.00125
D-Calcium Pantothenate	2.24
D-Glucose	2,700
Ferric Nitrate	0.05
Ferrous Sulfate, Heptahydrate	0.417
Folic Acid	2.66
Glycine	18.75
Hypoxanthine Sodium	2.385
L-Alanine	4.45
L-Arginine	147.5
L-Asparagine	7.5
L-Aspartic Acid	6.65
L-Cysteine	17.56
L-Cystine	31.285
L-Glutamine	365.1
L-Glutamic Acid	7.35
L-Histidine	31.48
L-Isoleucine	54.37
L-Leucine	58.95
L-Lysine	91.35
L-Methionine	17.24
L-Phenylalanine	35.48
L-Proline	17.25
L-Serine	26.25
L-Threonine	53.55
L-Tryptophan	9.02
L-Tyrosine	55.815
L-Valine	52.85
Linoleic Acid	0.044
Lipoic Acid	0.105
Magnesium Chloride	28.61
Magnesium Sulfate	48.85
Myo-Inositol	12.61
Niacinamide	2.0185
Phenol Red	8.1
Potassium Chloride	311.8
Putrescine, Dihydrochloride	0.08
Pyridoxine	2.031
Pyruvic Acid	110
Riboflavin	0.219
Sodium Chloride	6999.5
Sodium Phosphate, Dibasic	71
Sodium Phosphate, Monobasic	62.5
Thiamine	2.1685
Thymidine	0.365
Vitamin B12	0.68
Zinc Sulfate, Heptahydrate	0.4315

Supplemental Table 3.

Figure	n	Length	Purpose
1A	4-5	48 hours	A dose-response to determine if any concentration of progesterone reduces glycogen abundance in BUTE cells
1B-C	4	48 hours	Western blot to determine if any concentration of progesterone increases glycogen phosphorylase (PYG) protein level.
1D	6-7	48 hours	To determine if RU486 (nPR and PGRMC antagonist) could block the effect of progesterone on glycogen in BUTE cells
1E	7	48 hours	A dose-response to determine if any concentration of Org OD 02-0 (mPRα agonist) could mimic the effect of progesterone on BUTE cells
2	4	NA	IHC to determine if the the bovine endometrium expressed mPRs on days 1 and 11 of the reproductive cycle
3A	4	0.5 hours	To determine if progesterone or Org OD 02-0 could increase cAMP concentrations in BUTE cells at various time points.
3B	4	1 hour	
3C	4	24 hours	
3D	5	0-24 hours	To confirm that forskolin increased cAMP concentrations in BUTE cells
3E	6	48 hours	To confirm that increased cAMP concentrations did not result in reduced glycogen abundance in BUTE cells.
4A	5	NA	IHC to determine where pAMPK was localized in the bovine endometrium.
4B-C	5	24 hours	To confirm that progesterone could increase phosphorylation of AMPK in BUTE cells.
4D	6	48 hours	We tested the hypothesis that dorsomorphin (AMPK inhibitor) could block the effect of progesterone.
4E	5-6	48 hours	We tested the hypothesis that A-769662 (AMPK activator) would stimulate glycogenolysis, similar to progesterone.
5A	5-6	48 hours	To determine if D942 (which increases intracellular AMP concentrations) could stimulate glycogenolysis.
5B	4	48 hours	Determine if the effect of progesterone could be inhibitors by a glycogen phosphorylase inhibitor (GPI)
6A	4	NA	To Confirm that Ishikawa cells do not express the nPRs. We, and others, have previously shown this.
6B	6	48 hours	To determine if progesterone has similar effects in Ishikawa (human) cells as in BUTE cells
6C	5-6	48 hours	To determine if Org OD 02-0 has similar effects in Ishikawa (human) cells as in BUTE cells

Experiment (Figure), n, time, and purpose for all experiments. NA, not applicable.

Supplemental Table 4. Primary antibodies and conditions used for western blot (WB) and immunohistochemistry (IHC) analysis.

**Antigen**	**Catalog No**	**Technique**	**Dilution**	**Block**
mPRα (PAQR7)	HPA046936-100UL Sigma-Aldrich	IHC	1:100	Goat Serum
mPRδ (PAQR6)	HPA073505-100UL Sigma-Aldrich	IHC	1:100	Goat Serum
mPRε (PAQR9)	HPA052798-100UL Sigma-Aldrich	IHC	1:100	Goat Serum
pAMPKα	2535S Cell Signaling	WB	1:1000	BSA
		IHC	1:50	Goat Serum
AMPKα	5831S Cell Signaling	WB	1:1000	BSA
PYG	Ab231963 abcam	WB	1:500	BSA
GFP	2956S Cell Signaling	IHC	1:100/1:50	Goat Serum
Rabbit IgG	I-1000-5 Vector Laboratories	IHC	1:50	Goat Serum
nPR	A0321, ABclonal	WB	1:500	milk
α tubulin	2144S Cell Signaling	WB	1:500	BSA

Block for WB is 5% powdered milk or 5% BSA dissovled in TBS-T. Block for IHC is serum from 10% from appropiate species and 3% BSA dissovled in TBS.

Supplemental Table 5. Primer sequences used for mPRs analysis in BUTE and Ishikawa cells.

Species	Gene Name	Gene Symbol	Primer (5'→3')	Product Size (bp)	Accession Number
Cow	Membrane Progesterone Receptor α	*PAQR7*	F-GGCGCAAACTTGATCTTCATC<br>R-CTATTCCCATCTGTTCCCATCC	107	NM_001038553.1
	Membrane Progesterone Receptor β	*PAQR8*	F-TCTATGTCCTGTCCTCCATCA<br>R-CCACGAAGTAGAAGGTGTAGTG	99	NM_001101135.2
	Membrane Progesterone Receptor γ	*PAQR5*	F- CAAGACTCTGAGGAAGGAATGG<br>R-GAGGCTGAAGATGAGGCATAG	97	XM_002690486.5
	Membrane Progesterone Receptor δ	*PAQR6*	F-GGGAAGATGGTATCATGTCTGG<br>R-CAGATGTTGACGGTCTCGTT	100	NM_001046225.2
	Membrane Progesterone Receptor ε	*PAQR9*	F-CGATCAGGTGTACTACGTTGAG<br>R-GACCAGCAGCAGCATGTA	103	NM_001077951.1
	Progesterone Receptor Membrane Component 1	*PGRMC1*	F-CTGGGACTCTCAGTTCACTTTC<br>R-CTTCCGAGTGCTCTCATCTTT	115	NM_001075133.1
	Progesterone Receptor Membrane Component 2	*PGRMC2*	F-CGTATGAAGAAGCGGGACTT<br>R-TGGTCACGTCGAAGACTTTC	106	NM_001099060.1
Human	Membrane Progesterone Receptor α	*PAQR7*	F-CTAGGCAGGAGGTGAAACTTAG<br>R-CCGCATCCAGCAATGAAATC	98	NM_178422.6
	Membrane Progesterone Receptor β	*PAQR8*	F-GCAGAAGGGAAGAAGGTGTAAG<br>R-CTCTACCTCCGGTTCTCTCTTT	112	NM_133367.5
	Membrane Progesterone Receptor γ	*PAQR5*	F-TGTCCTCGCCTTTGCTTATC<br>R-GGTACGAGGTGGCTTCATTT	104	NM_017705.4

Progesterone Receptor Membrane Component 1	*PGRMC1*	F-TGAGGGAGGGACATACAGAATAG<br>R-GTACTGTTTCCTCGTGGTTCAG	85	NM_006667.5
Progesterone Receptor Membrane Component 2	*PGRMC2*	F-AGTCTTCGACGTGACCAAAG<br>R-GGCATCCCTACCAGCAAATA	76	NM_006320.6

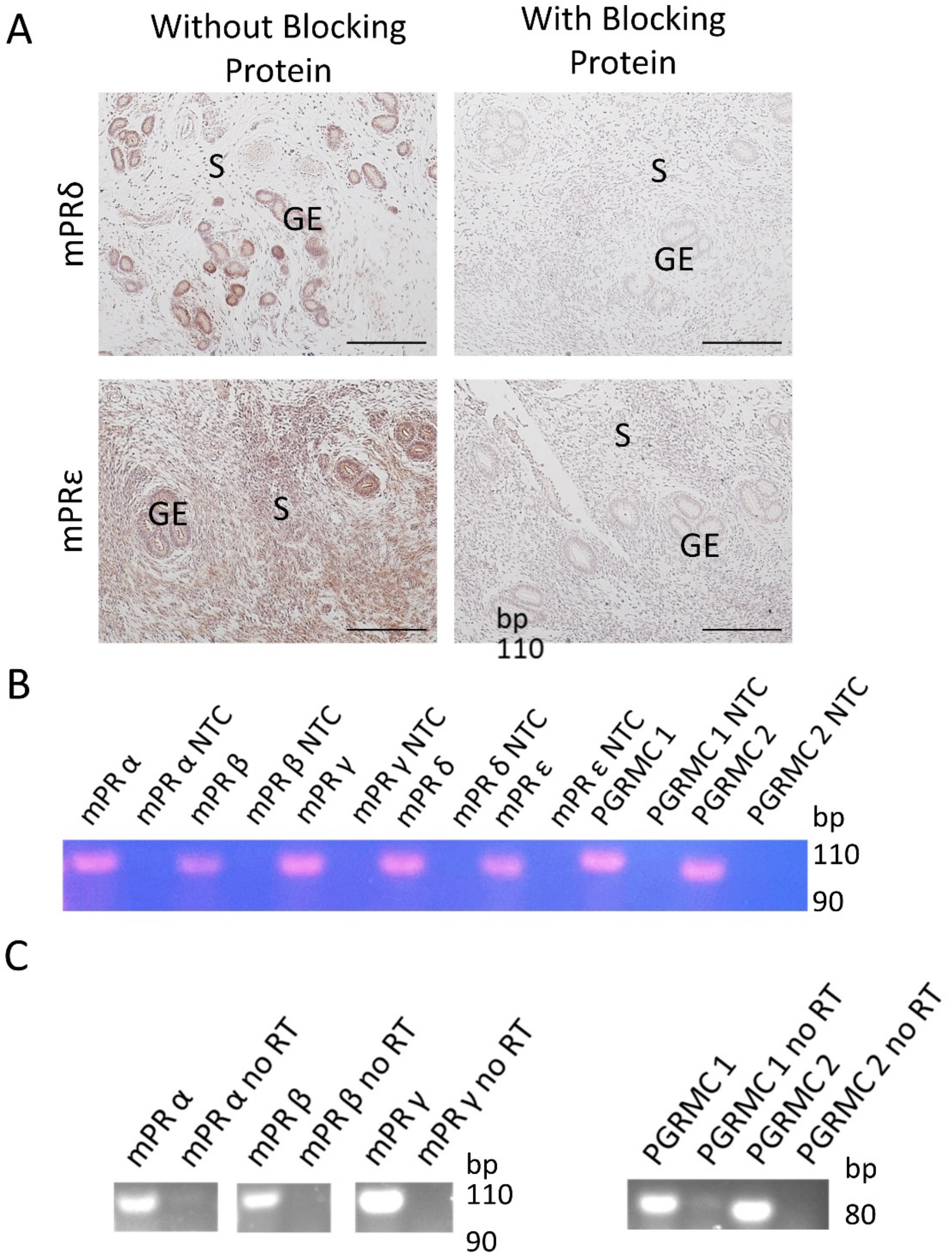

Supplemental Figure 1. A) Validation of antibodies for mPRδ and mPR ε using blocking peptides. B) RT-PCR showing that BUTE cells express all mPRs, PGRMC1, and PGRMC2. C) RT-PCR showing that Ishikawa cells express mPRα, mPRβ, mPRγ, PGRMC1, and PGRMC2. No RT is no reverse transcriptase negative control.
